# Multiple Episodes of Convergence in Genes of the Dim Light Vision Pathway in Bats

**DOI:** 10.1371/journal.pone.0034564

**Published:** 2012-04-11

**Authors:** Yong-Yi Shen, Burton K. Lim, He-Qun Liu, Jie Liu, David M. Irwin, Ya-Ping Zhang

**Affiliations:** 1 State Key Laboratory of Genetic Resources and Evolution, Kunming Institute of Zoology, the Chinese Academy of Sciences, Kunming, China; 2 Laboratory for Conservation and Utilization of Bio-resources, Yunnan University, Kunming, China; 3 Department of Natural History, Royal Ontario Museum, Toronto, Canada; 4 Graduate School of the Chinese Academy of Sciences, Beijing, China; 5 Department of Laboratory Medicine and Pathobiology, University of Toronto, Toronto, Canada; 6 Banting and Best Diabetes Centre, University of Toronto, Toronto, Canada; Lund University, Sweden

## Abstract

The molecular basis of the evolution of phenotypic characters is very complex and is poorly understood with few examples documenting the roles of multiple genes. Considering that a single gene cannot fully explain the convergence of phenotypic characters, we choose to study the convergent evolution of rod vision in two divergent bats from a network perspective. The Old World fruit bats (Pteropodidae) are non-echolocating and have binocular vision, whereas the sheath-tailed bats (Emballonuridae) are echolocating and have monocular vision; however, they both have relatively large eyes and rely more on rod vision to find food and navigate in the night. We found that the genes *CRX*, which plays an essential role in the differentiation of photoreceptor cells, *SAG*, which is involved in the desensitization of the photoactivated transduction cascade, and the photoreceptor gene *RH*, which is directly responsible for the perception of dim light, have undergone parallel sequence evolution in two divergent lineages of bats with larger eyes (Pteropodidae and Emballonuroidea). The multiple convergent events in the network of genes essential for rod vision is a rare phenomenon that illustrates the importance of investigating pathways and networks in the evolution of the molecular basis of phenotypic convergence.

## Introduction

Independent convergent evolution of phenotypic characters in response to similar selective pressures is not rare, however the molecular basis of these phenomena are poorly known [1], [2]. In previous studies, a single gene is often used to explain the phenotypic convergence of echolocation in mammals and dim-light vision in bats [3], [4], [5]. The genetic makeup of these phenotypic characters is very complex and undoubtedly many other genes are involved in both echolocation and vision. For example, dim-light vision requires a series of genes, not only the visual pigment genes, but also genes that are involved in the desensitization of the photoactivated pigments, photoreceptor development, and visual signal transduction. Multiple genes are essential for functional rod vision.

Bats are adapted to a nocturnal niche; however, their reliance on vision varies among species. Old World fruit bats (family Pteropodidae), for example, do not have laryngeal echolocation [6], and instead navigate largely by sight with larger eyes and binocular vision [7], [8], [9], [10]. Other types of bats have laryngeal echolocation, and in general have smaller eyes with monocular vision [11], [12], [13]. An exception to this are the sheath-tailed bats (Emballonuridae) who have relatively large eyes and appear to have a greater reliance on visual sight compared to most bats [14], [15]. The Emballonuridae with their relatively large superior colliculi resemble OW fruit bats in this respect [16], which may explain in part why both Emballonuridae and OW fruit bats both have well-developed visual systems [17]. The independent development of large eyes in Pteropodidae and Emballonuridae may reflect a functional convergence on the use of rod vision. Rod vision involves several processes, including light sensing, signal transduction, and interpretation in the brain cortex, among others, and thus many genes are involved [18], [19]. A previous study indicated that convergent evolution in rhodopsin (*RH1*), a rod vision gene that encodes the pigment directly responsible for the perception of dim light [20], had occurred in Pteropodidae and Emballonuridae [5]. Rod vision requires many other genes, such as *CRX*, which encodes the cone-rod homeobox protein that is a photoreceptor-specific transcription factor essential for the differentiation of photoreceptor cells [21]. *CRX* regulates the expression of many rod vision-specific genes [22], and mutations in this gene cause autosomal dominant cone-rod dystrophy [23], autosomal dominant retinitis pigmentosa [24] and Leber's congenital amaurosis [25], [26]. Another gene involved in rod vision is *SAG*, which encodes S-arrestin protein, a major soluble photoreceptor protein that is involved in the desensitization of the photoactivated transduction cascade. Mutations in *SAG* are associated with night blindness [27], [28]. While *RH1* is essential for perception in dim light, *CRX* and *SAG* are also of critical importance for the function of photoreceptor cells and the animal's ability to adapt to dim light.

In this study, we amplified and sequenced *CRX* and *SAG* genes from 38 individuals representing 29 species across the five major groups of bats (Emballonuroidea, Noctilionoidea, Pteropodidae, Rhinolophoidea, and Vespertilionoidea). Similar to previous findings with *RH1* [5], we found evidence supporting convergent evolution in both *CRX* and *SAG*, providing a rare example of multiple events of convergent evolution occurring in parallel in interrelated genes, suggesting that multiple changes are involved in the network of genes necessary for rod vision to generate the complex molecular and phenotypic convergences.

## Results

### The parallel sequence evolution of *CRX* genes

*CRX* genes were amplified from 38 individuals representing 29 species of bats. The aligned nucleotide sequence was 861 base pairs (bp) in length, of which 287 were variable and generated 83 sites with amino acid variation (Figure S1). The amplified *CRX* sequence corresponds to bases 25 to 879 of the 897 base coding sequence of the human gene. No insertion/deletion mutations or change that resulted in a stop codon were found in any of the sequences, suggesting that all of the bats have a functional *CRX* gene.

Phylogenetic analyses of the aligned *CRX* nucleotide sequences (861 bp) with Bayesian, Maximum Likelihood and Neighbor-joining methods resulted in consistent trees that were congruent with the best-supported species tree [29], [30] (Figure S2). While Pteropodidae and Emballonuridea are two divergent lineages of bats, the phylogenetic tree generated from amino acid sequence data placed Emballonuroidea and Pteropodidae together (Figure S3). If only nonsynonymous nucleotide changes were used to reconstruct the topology of bats (Figure S4), Pteropodidae was found not to group with Rhinolophoidea (as expected from the nucleotide sequence phylogeny – Figure S2), but instead had a closer relationship with Emballonuroidea and Vespertilionoidea. The bootstrap support values for these relationships in the amino acid and nonsynonymous trees were low, which is most likely due to the small number of nonsynonymous and amino acid substitutions that can be used to reconstruct the topologies.

**Figure 1:**
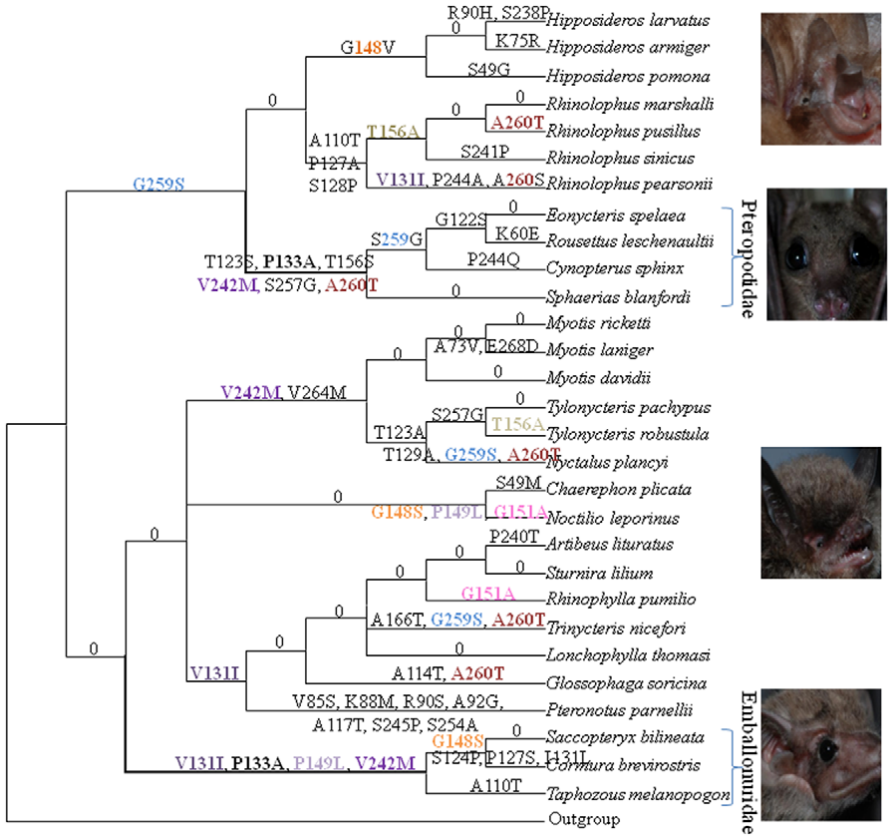
Convergent evolution of the *CRX* gene in bats based on a tree derived from the Bayesian analysis of nucleotide sequences. Numbers and symbols above the branches are the positions and amino acid replacements. Sequences at the internal nodes were reconstructed by the Maximum Likelihood method in PAML.

To further examine the evolution of *CRX*, ancestral *CRX* sequences, at the internal nodes of the species tree, were reconstructed and the changes that occurred on each lineage were inferred. Parallel changes at amino acid positions 133 (P133A marked in bold black) and 242 (V242M marked in purple) were found in both Pteropodidae and Emballonuroidea (Figure 1). If these two amino acid sites were excluded from the phylogenetic analysis, then the phylogeny was in accord with the nucleotide sequence and the best-supported species tree. Using a statistical test [31] the two branches were shown to contain significantly larger number of parallel evolving sites than expected (*P*<0.001). Of the two sites that show parallel changes, amino acid 133 was found to be perfectly conserved in all other mammals examined as proline, except in the bat species in Pteropodidae and Emballonuridea, while the amino acid site 242 showed greater variation (Figure S1).

The maximum likelihood estimate of the average ratio of nonsynonymous to synonymous substitution rate (*Ka/Ks*) was 0.0754 (M_0_ model) (Table S1). When Emballonuroidea and Pteropodidae were set as independent foreground lineages, and tested for selection using PAML, we failed to find any signal for positive selection. However, if the two branches were set as a combined foreground lineage, then the branch_site model indicated marginal evidence that these two branches had experienced positive selection (the LRT test statistic, 2⊿l = 3.516, *P* = 0.06, see Table S1), with the sites 133P (pp = 0.888) and 242V (pp = 0.976) on these lineages being the positively selected sites.

### The parallel sequence evolution of *SAG*

*SAG* genes were successfully amplified from the 25 individuals representing 18 species of bats. The amplified *SAG* sequence corresponds to bases 181 to 951 of the 1218 base coding sequence of the human gene. No insertion/deletion mutations or changes that result in stop codons were found in any of the sequences, suggesting that all bats have a functional *SAG* gene. The aligned *SAG* nucleotide sequences were 771 bp in length, including a 3 bp insertion, of which 210 were variable and generated 55 sites with amino acid variations (Figure S5). Phylogenetic trees generated from the nucleotide (771 bp) and amino acid (257 sites) sequences of the aligned *SAG* gene by multiple methods resulted in trees that were congruent with the best-supported phylogeny of bats generated from other data sources [29], [30] (Figure S6).

**Figure 2:**
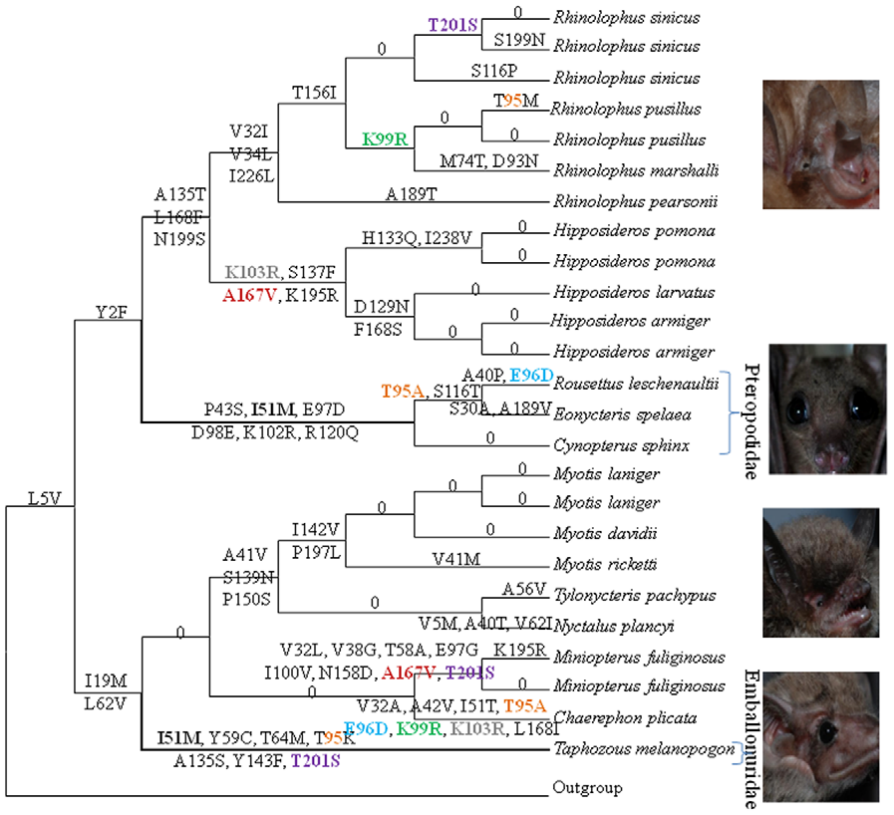
Convergent evolution of the *SAG* gene in bats based on a tree derived from the Bayesian analysis of nucleotide sequences. Numbers and symbols above the branches are the positions and amino acid replacements. Sequences at internal nodes were reconstructed by the Maximum Likelihood method in PAML.

Ancestral *SAG* sequences for the internal nodes of the species tree were reconstructed and amino acid substitutions were inferred onto each lineage. Pteropodidae and Emballonuroidea were both found to share an I51M amino acid replacement (marked in bold black, Figure 2) at a site that is conserved among examined mammals (Figure S5). The probability that this parallel evolutionary change occurred by random on these two branches was significantly rejected (*P* = 0.011) by a statistical test [31]. Additional parallel replacements were observed between different bat lineages (e.g., T201S, K99R, T95A, and E96D), however they occurred on other branches that had no obvious shared morphological or ecological similarity and as these were sites that are not conserved within mammals, thus the parallel changes at these sites may not have functional importance.

The maximum likelihood estimate of the average ratio of the rates of nonsynonymous to synonymous substitution (*Ka/Ks*) was 0.0933 (M_0_ model). Both the branch model and branch site model in PAML failed to detect any significant signal for positive selection on the lineages for the common ancestor of all bats, Emballonuroidea bats or Pteropodidae bats (Table S2).

## Discussion

The development of morphological characters is very complex and typically involves a series of genes. Eye development is an example that probably requires the actions of thousands of genes [32]. The fact that large numbers of genes are involved, indicates that the convergent evolution of these complex phenotypic characters, whether vision or echolocation, likely cannot be fully explained by the evolution of a single gene. It would appear that many genes, driven by similar selective pressures, are required for functional convergence. In this study, we tested several genes that are involved in different aspects of rod vision function to determine if similar patterns of evolution occurred to them during the evolution of a morphological character – evolving larger eyes, which may reflect a greater reliance on rod vision.

Mammals have two distinct types of photoreceptors, rods and cones, which display important differences in their sensitivity to light intensity and ranges in light wavelength photosensitivity. Rods have a high sensitivity to light and thereby mediate nighttime vision when there are few photons. In contrast, cone photoreceptors serve for daylight vision when photons are plentiful [33]. In general, nocturnal mammals have relatively larger eyes than diurnal species in order to maximize their visual sensitivity [34], [35]. Although bats are nocturnal, most species navigate using laryngeal echolocation, with a limited need for vision, and thus have characteristically small eyes [36]. Bats from the family Pteropodidae, however, do not have laryngeal echolocation, and therefore generally navigate by sight, and thus have larger eyes [7], [8], [9], [10]. While bats from the family Emballonuridae do have laryngeal echolocation, they also have relatively big eyes and are more active at dusk compared to other echolocating bats [14]. The independent development of large eyes may reflect a shared greater functional reliance on rod vision.

*CRX* is a developmental regulatory gene associated with the differentiation of photoreceptor cells involved in rod vision development while *SAG* is a component of the photo-signaling cascade and functions in the desensitization of the photoactivated transduction cascade. Both genes are involved in distinct but important roles of rod cells. When the evolution of the *CRX* and *SAG* protein sequences was examined in bats, we identified two parallel changes in the amino acid sequence of *CRX* and one parallel amino acid change in *SAG* that are shared by Emballonuroidea and Pteropodidae. The amino acid sites involved in these parallel changes in these two genes are in general conserved in the sequences for these two genes in other mammals (shown in Figures S1 and S5). Previously we had found convergent evolution in *RH1* [5], a rod cell gene directly responsible for the perception of dim light [20]. We hypothesize that these multiple instances of parallel sequence evolution between Emballonuroidea and Pteropodidae bats reflect parallel functional convergence for dim light vision and also reflect the complex genetic mechanisms that are required for this phenotypic convergence.

The three instances of parallel evolution in the *CRX* and *SAG* genes discussed above were not the only instances of convergent amino acid substitutions observed in these genes in bats. Parallel changes in the *CRX* gene was observed at amino acid position 259 (G259S, marked in purple in Figure 1) on four divergent branches and at position 260 (A260T, marked in coffee in Figure 1) five times. These multiple changes, which were observed on multiple branches, imply that during the long history of bats, *CRX* may have been prone to convergence, possibly due to ecological specializations (i.e., to photic environments). Alternatively, since neither of these two sites are well conserved within *CRX* gene sequences mammals in general, they may simply reflect changes that are tolerated, and these two amino acid states may have no functional difference in bats [5]. Similarly, except the parallel site (I51M) that occurred on Emballonuroidea and Pteropodidae, a few other sites showed parallel changes in other branches, however these sites were also not conserved within mammals and these changes may have no functional consequence.

Old World fruit bats (Pteropodidae) do not echolocate but instead rely on their other senses, such as vision, to find food and navigate at night. Although insect-feeding bats rely largely on the key innovation of echolocation to find prey and navigate in close quarters at night, vision has been retained and serves as an important complement to echolocation [14], [37], [38]. In most echolocating bats, vision is predominantly used only for long-range navigation, where echolocation is less effective [14]. Our analysis found that three rod vision genes (*CRX*, *SAG* and *RH1*) have each experienced strong purifying selection in all bats, reflecting a common need for rod vision in all bats. Bats from two families, Pteropodidae and Emballonuroidea, rely on vision to a greater extent than other bats [14], however, a significant signal for positive selection was not detected on either of these two branches, instead we found that the sequences of these three vision genes had undergone parallel sequence evolution. The failure to detect positive selection may reflect the difficulty of obtaining statistically significant evidence by these methods despite the presence of positively selected amino acid substitutions [39].

Non-neutral convergent evolution of morphological characters should cause bias in phylogenetic inference [40], [41], [42]. Phylogenetic analyses of *Prestin* [3], [4], [43], *CRX* (this study) and *RH1* [5] using protein sequences or nonsynonymous sites fail to recover the expected species tree due to convergent evolution. However, we failed to detect evidence for convergence in the *SAG* gene using phylogenetic approaches, and this difference is likely due to the number of changes that occurred in the sequences. Many amino acid substitutions occurred in the *SAG* sequences on both branches of the Old World fruit bats and sheath-tailed bats, thus the single parallel amino acid site did not result in a “false” tree, as the larger number of other changes overwhelmed this signal, and instead support a the species phylogeny consistent with that generated by other types of data. Convergent evolution, however, was detected in the *SAG* sequences when ancestral sequences were reconstructed for the internal nodes.

Vision plays a basic role in the survival of most animals. Bats are nocturnal, however Old World fruit bats, and sheath-tailed bats may use eyesight to navigate and, compared with other insect-feeding bats that rely on echolocation, have larger eyes. Our study showed, in addition to our previous finding of the *RH1* gene [5], that the genes *CRX* and *SAG* have undergone parallel sequence evolution in two divergent lineages of bats with larger eyes (Pteropodidae and Emballonuroidea). These parallel changes in these three genes in two branches of bat phylogeny likely result from the common selection for amino acid-altering mutations [3] that are beneficial for dim light vision. The finding of multiple convergences in the network of genes essential for rod vision in bats reflect the complex mechanisms that drove the adaptation to dim light environments during the successful radiation of the second most diverse order of mammals as they exploited the aerial nocturnal niche. Similarly, recent studies have shown that at least two genes, through adaptive evolution, contributed to the evolution of echolocation [3], [43], [44], [45]. Our study demonstrates that greater attention should be focused on the molecular evolution of pathways and networks for a better understanding of phenotypic convergence.

## Materials and Methods

### Ethics Statement

All research involving animals used in this study followed the guidelines of the by-laws on experimentation on animals, and was approved by the Ethics and Experimental Animal Committee of the Kunming Institute of Zoology, Chinese Academy of Sciences (KIZ_YP201002).

### Source of data and primary treatments

*CRX* and *SAG* gene sequences of the little brown bat (*Myotis lucifugus*), flying fox (*Pteropus vampyrus*), cow and dog were downloaded from the Ensembl database. Sequences of these genes were aligned using CLUSTALX 1.81 [46]. Gene-specific primers were designed based on conserved regions. Fresh eye tissue was available for Old-World bat species, thus RNA was isolated, converted to cDNA and used as template to amplify the *CRX* and *SAG* coding sequences. RNA samples were not available for New-World bat species, thus genomic sequences were amplified with exon-specific primers (Table S3 and Table S4). Genes for *CRX* and *SAG* were amplified from a total of 38 individuals, representing 29 species of bats, in this study, and were analyzed together with other sequences that were available from GenBank and Ensembl. For the isolation of RNA, 40 bat individuals (listed in Table S5) were sacrificed followed the guidelines of the by-laws on experimentation on animals, and was approved by the Ethics and Experimental Animal Committee of the Kunming Institute of Zoology, Chinese Academy of Sciences, and their eyes were rapidly excised and frozen in liquid nitrogen. Total RNA was isolated from the eyes using the RNAiso™ Plus Kit (Takara, China), and stored at −80°C. cDNA for RT-PCR was generated from 2 µg RNA using the PrimeScript™ RT-PCR Kit (Takara, China). Total genomic DNA was extracted using a standard 3-step phenol/chloroform extraction method [47]. *CRX* and *SAG* genes were amplified from the cDNA or total DNA using gene-specific primers (Tables S3 and S4). PCR amplifications were carried out using the following touchdown program: 95°C 4 min, 20 cycles of 94°C denaturation 1 min, 60–50°C annealing (1 min; −0.5°C/cycle) or 63°C, 72°C extension 1 min, and finally 15 cycles of 94°C 1 min, 50°C 1 min, 72°C 1 min. PCR products were cleaned using the Watson PCR Purification Kits (Watson BioTechnologies, Shanghai). Each PCR product was sequenced at least three times on an ABI 3730 Sequencer (Applied Biosystems, Foster, CA, USA) using the ABI PRISM BigDye Terminator v3.0. DNA sequences were edited using DNAstar Seqman software (DNASTAR Inc., Madison, WI, USA). The new *CRX* and *SAG* sequences were deposited into GenBank (Accession numbers HQ651094–HQ651149, JF831422–JF831446).

### Phylogenetic and Molecular Evolutionary Analyses

For each gene, nucleotide sequences were translated into amino acid sequences and aligned using CLUSTALX 1.81 [46] as a guide for the alignment of the nucleotide sequences for evolutionary analyses. The best fit models for nucleotide and amino-acid substitutions were determined by ModelTest [48] and ProtTest v2.4 [49], respectively, under the Akaike information criterion. The computer algorithm PhyML [50] was used to construct maximum-likelihood (ML) phylogenies of the nucleotide and amino-acid data under their best-fitting models. Bayesian inference (BI) and neighbor-joining (NJ) phylogenies were constructed using MrBayes [51] and MEGA 4 [52], respectively.

We used the Li-Wu-Luo method [53] to reconstruct a NJ tree based on synonsymous and nonsynonymous sites. Each site in a codon is allocated to a 0-fold, 2-fold or 4-fold degenerate category. For computing distances, all 0-fold and two-thirds of the 2-fold sites are considered nonsynonymous, whereas one-third of the 2-fold and all of the 4-fold sites are considered synonymous changes.

Tests for selection and ancestral sequence reconstruction were carried out using the Codeml program implemented in PAML [54], [55]: (1) one-ratio model, which assumes an identical ω value for all branches, where ω is the ratio of nonsynonymous to synonymous substitution rates; (2) a free-ratio model, assuming an independent ω values for each branch, to provide a rough measure of the selective pressure on each branch; (3) two-ratio model and (4) branch-site model were used to determine whether these genes have undergone positive selection on a foreground branch; (5) site models: the neutral model (M1a) estimates two ω values (0<ω_0_<1, ω_1_ = 1); the positive selection model (M2a) adds an extra ω value to M1a; M8 (β &ω model) takes into account the possibility of positively selected (PS) sites; and M8a is the null model of M8. Bayes Empirical Bayes (BEB) analysis was used to calculate the Bayesian posterior probability of PS sites. Finally, LRT statistics were calculated between the following model pairs: (1) the two-ratio model vs. the one-ratio model were compared to test whether the ω ratio is significantly different from that of other mammals; (2) test 1 (branch-site model vs. site model M1a) and test 2 (branch-site model vs. branch-site model with fixed ω_1_ = 1) for branch-site model [56] were conducted; (3) M1a vs. M2a and M8 vs. M8a were compared to examine possible positive selection sites. In the previous cases, twice the difference in log-likelihood values (2ΔlnL) between the two models was calculated following a chi-squared (χ2) distribution with the degrees of freedom equaling the difference in the number of parameters estimated for the model pairs.

## Supporting Information

Figure S1 **Amino acid replacements in the** ***CRX*** **gene sequences of bats.** The asterisk is the site of the convergent amino acid replacement P133A.
(TIF)

Figure S2 **Topology based on the nucleotide sequences of** ***CRX*** **.** Numbers above the branches are Bayesian posterior probabilities, and numbers below the branches are the ML and NJ bootstrap values.
(TIF)

Figure S3 **Topology based on amino acid sequences of** ***CRX*** **.** Numbers above the branches are the Bayesian posterior probabilities.
(TIF)

Figure S4 **NJ tree based on the nonsynonsymous sites of the** ***CRX*** **gene.** Numbers above the branches are the NJ bootstrap values.
(TIF)

Figure S5 **Amino acid replacements in the** ***SAG*** **gene sequences of bats.** The asterisk is the site of the amino acid replacement I51M.
(TIF)

Figure S6 **Topology of** ***SAG*** **.** (A) Topology based on the nucleotide sequences of *SAG*. Numbers above the branches are the Bayesian posterior probabilities, and below are the ML and NJ bootstrap values. (B) Topology based on amino acid sequences of *SAG*. Numbers above the branches are the Bayesian posterior probabilities.
(TIF)

Table S1 **Analyses of the selective pressure on the** ***CRX*** **gene of bats.**
(DOC)

Table S2 **Analyses of the selective pressure on the** ***SAG*** **gene of bats.**
(DOC)

Table S3 **Primers used for amplifying and sequencing** ***CRX*** **genes in bats.**
(DOC)

Table S4 **Primers used for amplifying and sequencing** ***SAG*** **genes in bats.**
(DOC)

Table S5 **Species and their accession numbers of** ***CRX*** **and** ***SAG*** **genes used in this research.**
(DOC)
