# Supplementary figures and images for: Multiple Episodes of Convergence in Genes of the Dim Light Vision Pathway in Bats
Source: PLoS One. 2012 Apr 11;7(4):e34564. doi: 10.1371/journal.pone.0034564 (PMC3324491; doi:10.1371/journal.pone.0034564)

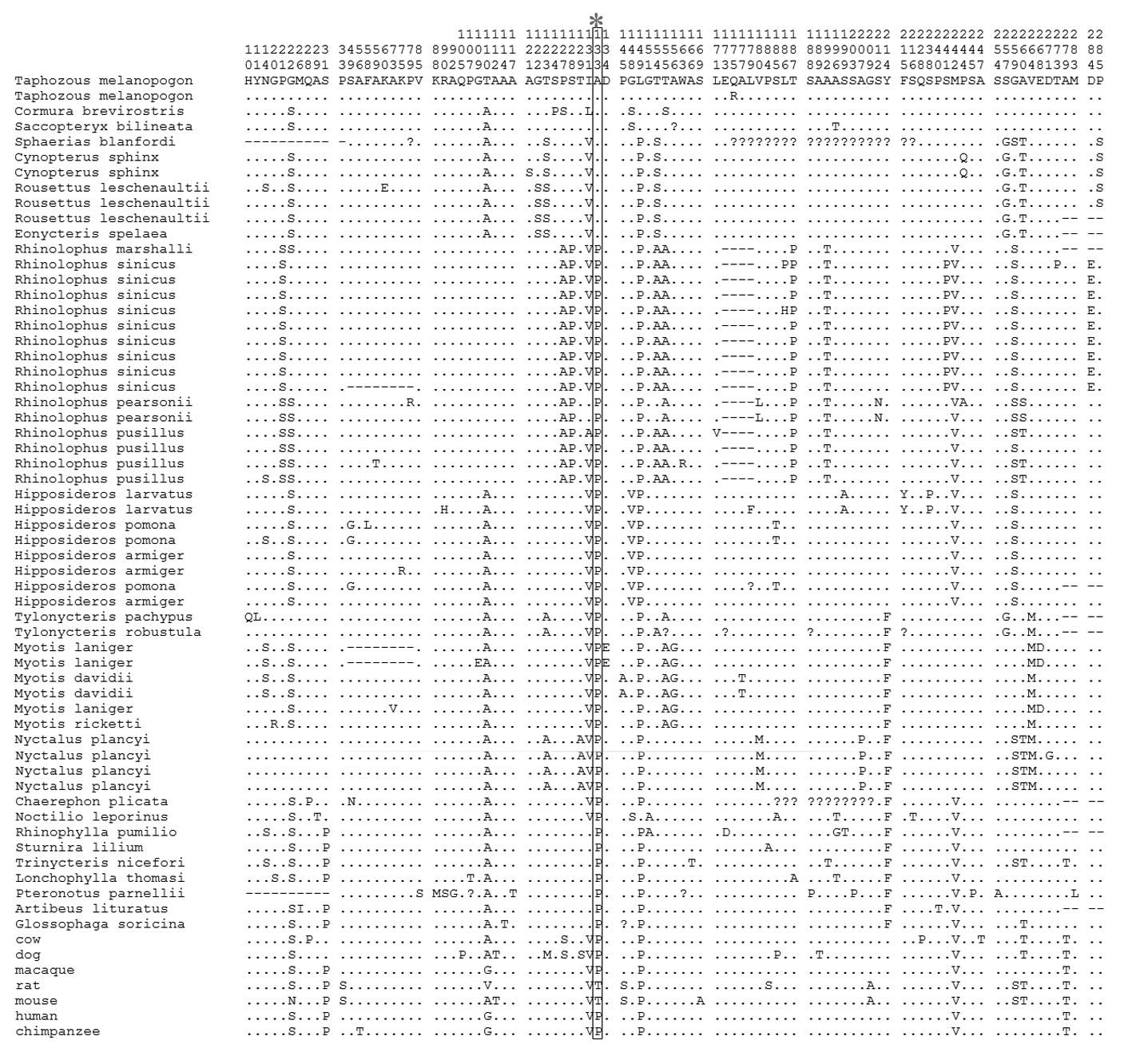

Supplement: Figure S1 — Amino acid replacements in the CRX gene sequences of bats. The asterisk is the site of the convergent amino acid replacement P133A. (TIF) [file pone.0034564.s001.tif]

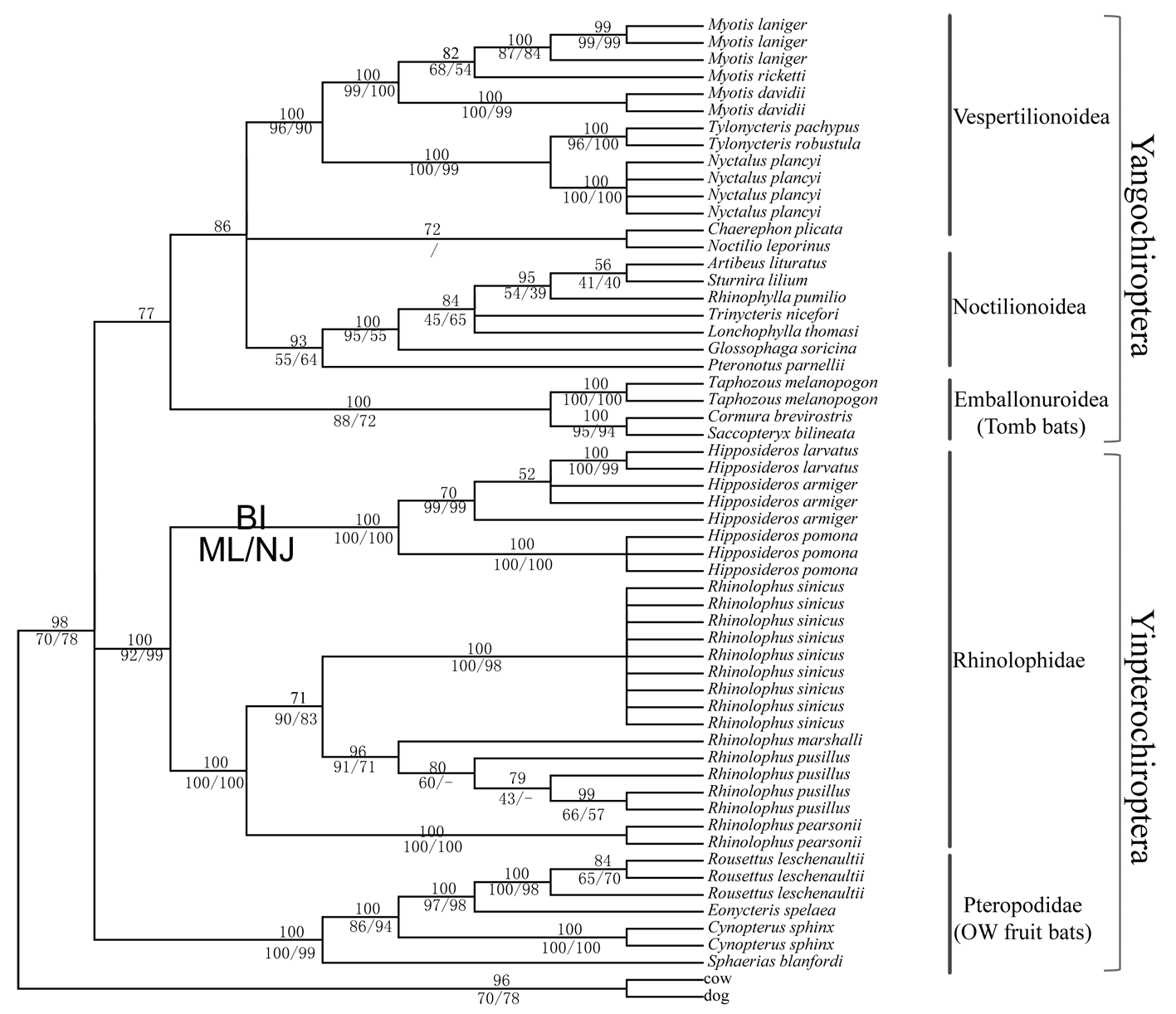

Supplement: Figure S2 — Topology based on the nucleotide sequences of CRX . Numbers above the branches are Bayesian posterior probabilities, and numbers below the branches are the ML and NJ bootstrap values. (TIF) [file pone.0034564.s002.tif]

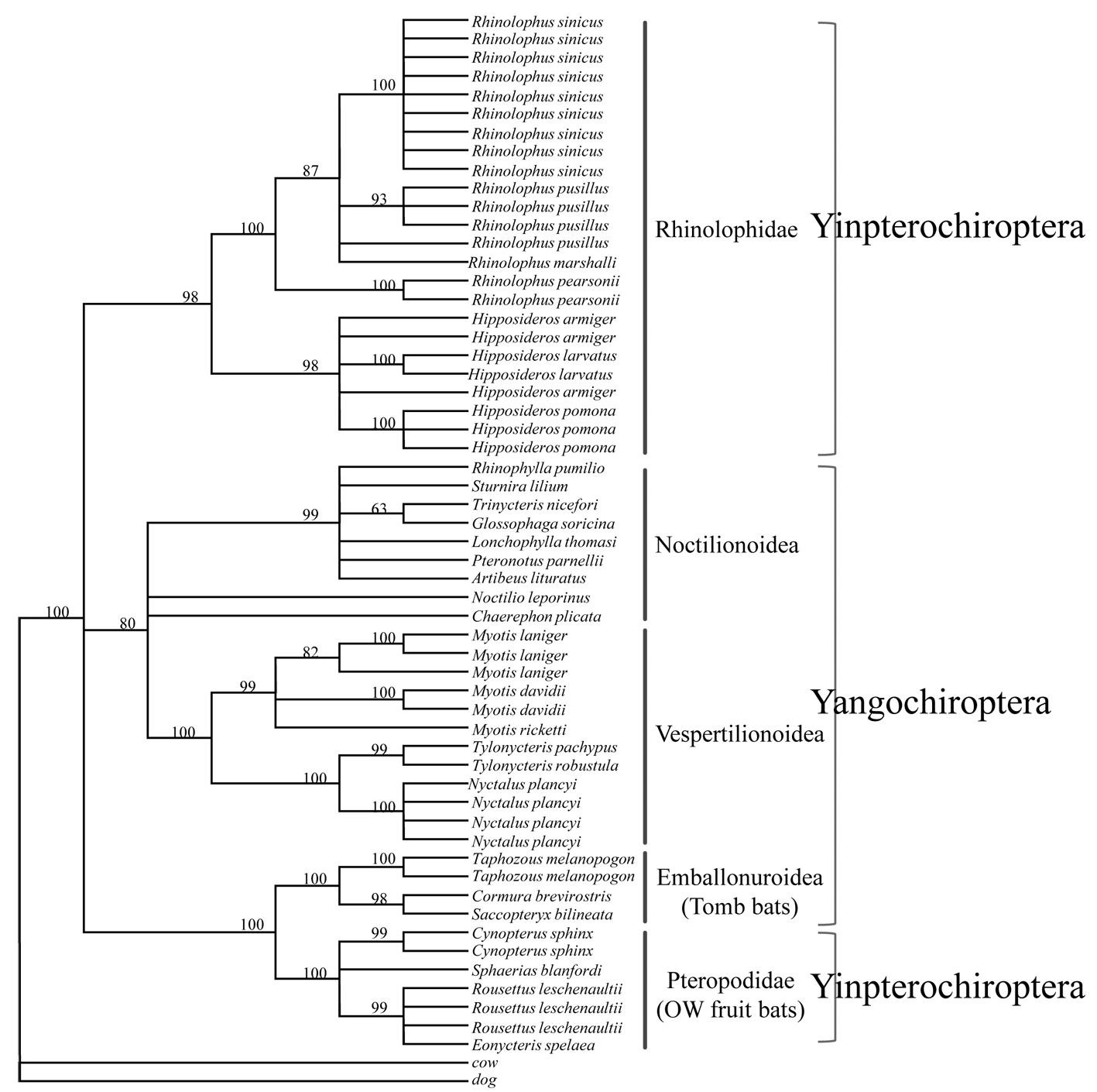

Supplement: Figure S3 — Topology based on amino acid sequences of CRX . Numbers above the branches are the Bayesian posterior probabilities. (TIF) [file pone.0034564.s003.tif]

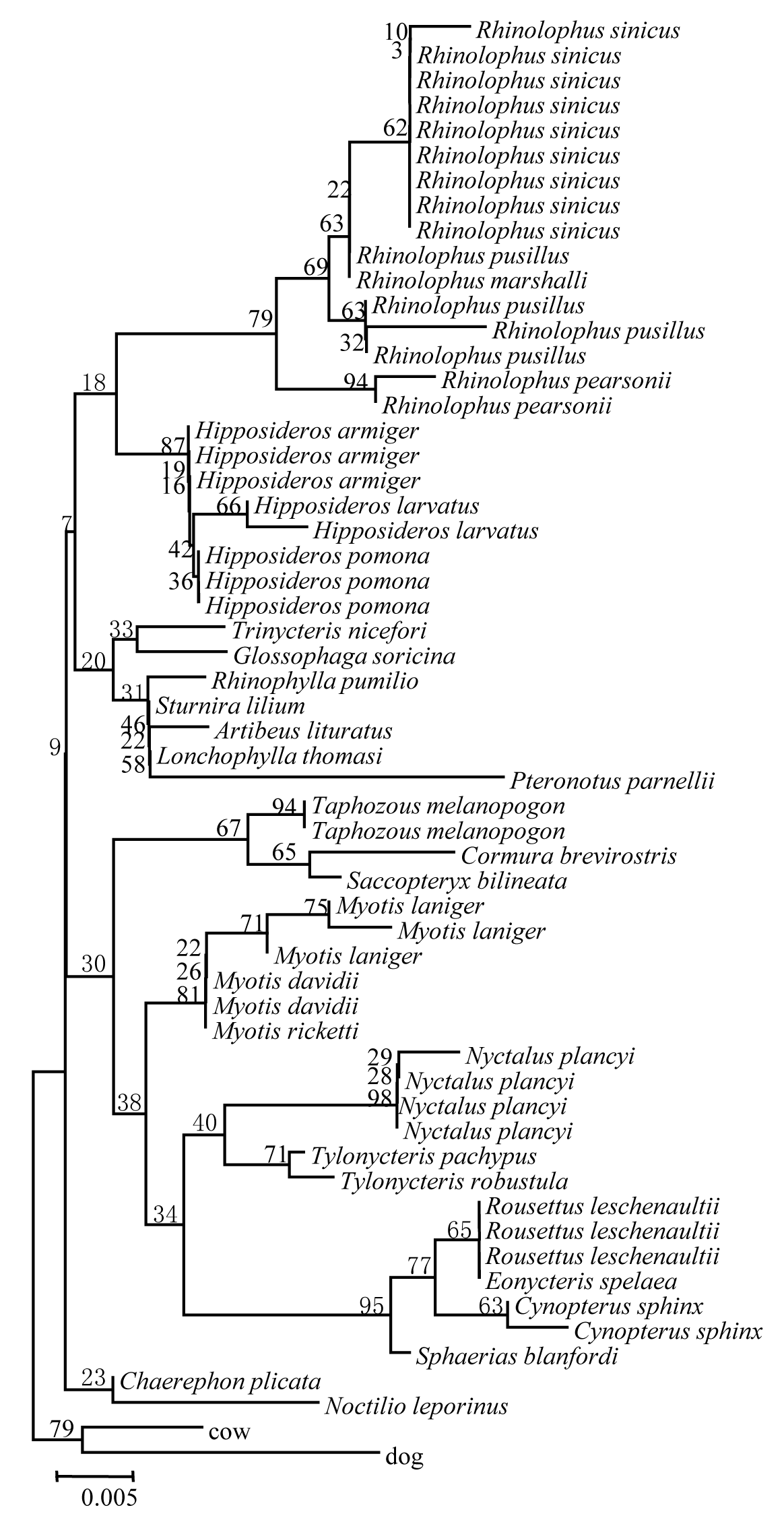

Supplement: Figure S4 — NJ tree based on the nonsynonsymous sites of the CRX gene. Numbers above the branches are the NJ bootstrap values. (TIF) [file pone.0034564.s004.tif]

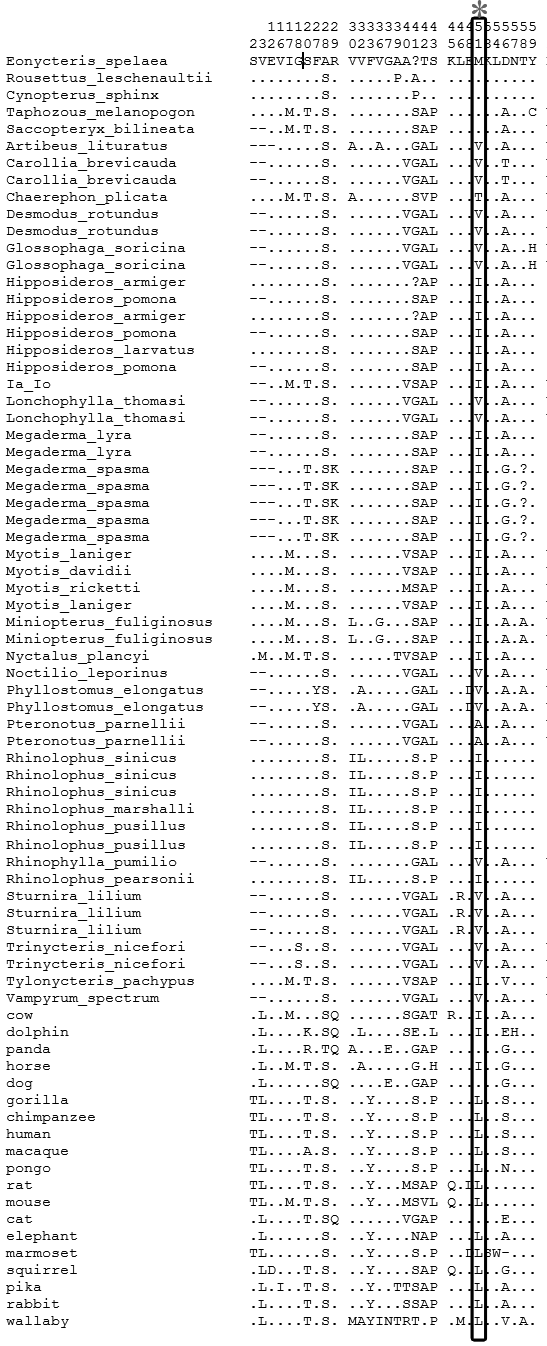

Supplement: Figure S5 — Amino acid replacements in the SAG gene sequences of bats. The asterisk is the site of the amino acid replacement I51M. (TIF) [file pone.0034564.s005.tif]

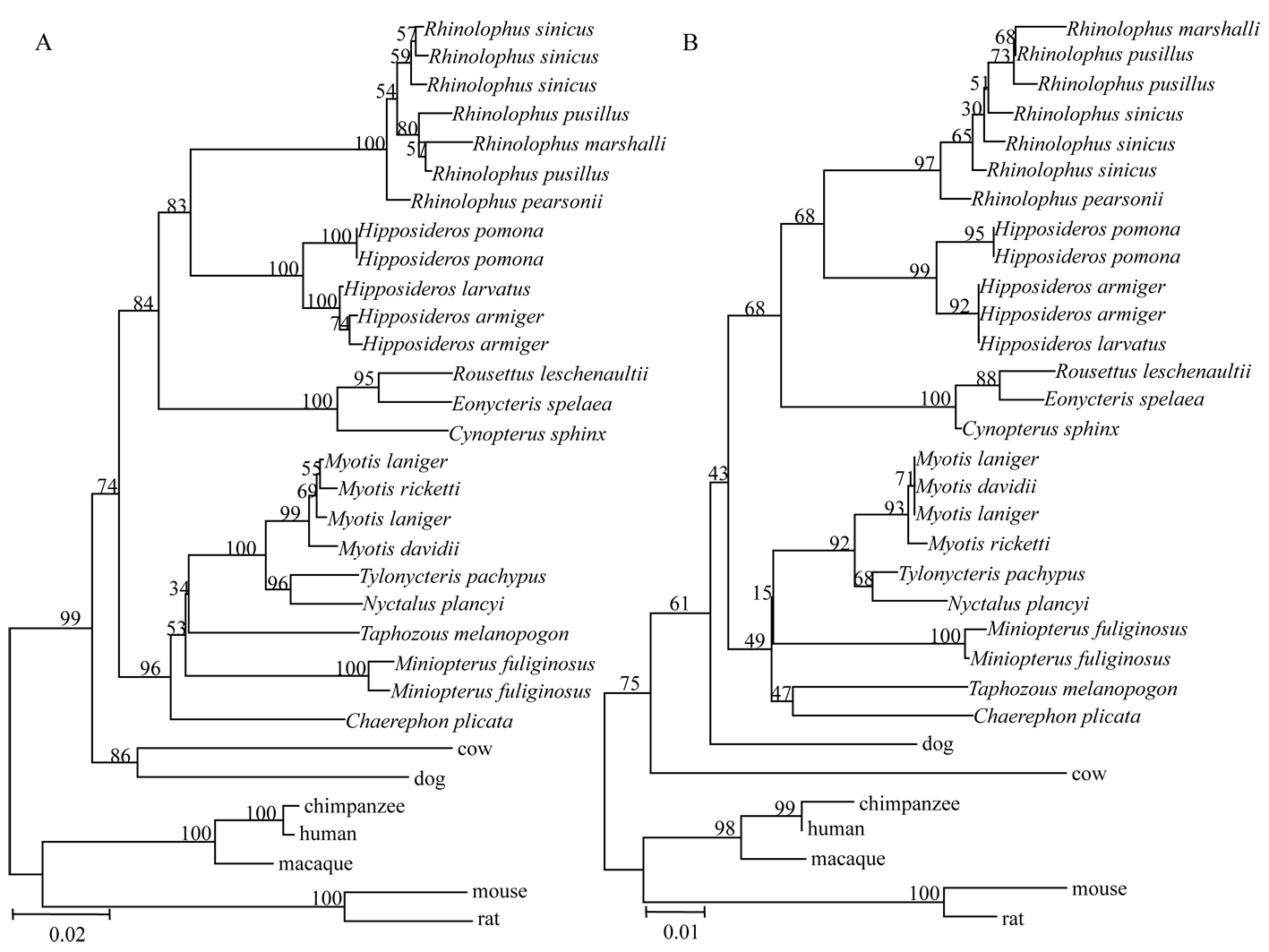

Supplement: Figure S6 — Topology of SAG . (A) Topology based on the nucleotide sequences of SAG. Numbers above the branches are the Bayesian posterior probabilities, and below are the ML and NJ bootstrap values. (B) Topology based on amino acid sequences of SAG. Numbers above the branches are the Bayesian posterior probabilities. (TIF) [file pone.0034564.s006.tif]
