# Supplementary material for: Multiple Episodes of Convergence in Genes of the Dim Light Vision Pathway in Bats
Source: PLoS One. 2012 Apr 11;7(4):e34564. doi: 10.1371/journal.pone.0034564 (PMC3324491; doi:10.1371/journal.pone.0034564)
Supplement: Table S1 — Analyses of the selective pressure on the CRX gene of bats. (DOC) [file pone.0034564.s007.doc]

Table S1: Analyses of the selective pressure on the *CRX* gene of bats.

| **Model** | **P** | **Ln L** | **Estimates of parameters** |  |
| --- | --- | --- | --- | --- |
| **M_0_: one ratio** | 111 | -2531.95 | ω_0_= 0.0754 |  |
| **Two ratios:** | | | |  |
| **The common ancestor of bats** | 112 | -5915.29 | ω_b_=0.0885, ω_0_= 0.0661 |  |
| **The common ancestor of bats**  **ω_b_=1** | 111 | -5917.43 | ω_b_=1.0000, ω_0_= 0.0656 |  |
| **Pteropodidae** | 112 | -5915.15 | ω_m_= 0.0861, ω_0_= 0.0656 |  |
| **Pteropodidae ω_m_=1** | 111 | -5931.77 | ω_m_=1.0000, ω_0_= 0.0649 |  |
| **Emballonuridae** | 112 | -5914.37 | ω_t_=0.1664, ω_0_=0.0652 |  |
| **Emballonuridae ω_t_=1** | 111 | -5918.13 | ω_t_=1.0000, ω_0_=0.0651 |  |
| **Emballonuridae and Pteropodidae** | 112 | -5914.53 | ω_tm_=0.1051, ω_0_=0.0645 |  |
| **Emballonuridae and Pteropodidae**  **ω_tm_=1** | 111 | -5934.71 | ω_tm_=1.0000, ω_0_=0.0638 |  |
| **Branch-site models** | | | |  |
| **Pteropodidae** | 114 | -5888.76 | site class 0 1 2a 2b  proportion 0.96175 0.03784 0.00039 0.00002  background ω 0.04984 1.00000 0.04984 1.00000  foreground ω 0.04984 1.00000 1.00000 1.00000 |  |
| **Pteropodidae ω_m_=1** | 113 | -5888.76 | site class 0 1 2a 2b  proportion 0.96175 0.03784 0.00039 0.00002  background ω 0.04984 1.00000 0.04984 1.00000  foreground ω 0.04984 1.00000 1.00000 1.00000 |  |
| **Emballonuridae** | 114 | -5888.65 | site class 0 1 2a 2b  proportion 0.93339 0.03633 0.02914 0.00113  background ω 0.04967 1.00000 0.04967 1.00000  foreground ω 0.04967 1.00000 1.00000 1.00000 |  |
| **Emballonuridae ω_t_=1** | 113 | -5888.65 | site class 0 1 2a 2b  proportion 0.93340 0.03633 0.02914 0.00113  background ω 0.04967 1.00000 0.04967 1.00000  foreground ω 0.04967 1.00000 1.00000 1.00000 |  |
| **Emballonuridae and Pteropodidae** | 114 | -5885.25 | **site class 0 1 2a 2b**  **proportion 0.95725 0.03311 0.00932 0.00032**  **background ω 0.04941 1.00000 0.04941 1.00000**  **foreground ω 0.04941 1.00000 11.94062 11.94062** | 2⊿l = 3.516  *P*=0.06 |
| **Emballonuridae and Pteropodidae**  **ω_tm_=1** | 113 | -5887.01 | site class 0 1 2a 2b  proportion 0.92820 0.03388 0.03658 0.00134  background ω 0.04878 1.00000 0.04878 1.00000  foreground ω 0.04878 1.00000 1.00000 1.00000 |
