# Supplementary material for: Multiple Episodes of Convergence in Genes of the Dim Light Vision Pathway in Bats
Source: PLoS One. 2012 Apr 11;7(4):e34564. doi: 10.1371/journal.pone.0034564 (PMC3324491; doi:10.1371/journal.pone.0034564)
Supplement: Table S2 — Analyses of the selective pressure on the SAG gene of bats. (DOC) [file pone.0034564.s008.doc]

Table S2: Analyses of selective pressure on the *SAG* gene of bats.

| **Model** | **P** | **Ln L** | **Estimates of parameters** |
| --- | --- | --- | --- |
| **M_0_: one ratio** | 64 | -3941.44 | ω_0_=0.0933 |
| **Two ratios:** | | | |
| **The common ancestor of bats** | 65 | -3941.20 | ω_b_=0.0470, ω_0_=0.0942 |
| **The common ancestor of bats ω_b_=1** | 64 | -3946.28 | ω_b_=1.0000, ω_0_=0.0924 |
| **Pteropodidae** | 65 | -3940.76 | ω_m_= 0.0575, ω_0_= 0.0959 |
| **Pteropodidae ω_m_=1** | 64 | -3964.51 | ω_m_=1.0000, ω_0_= 0.0936 |
| **Emballonuridae** | 65 | -3940.87 | ω_t_= 0.1497, ω_0_= 0.0913 |
| **Emballonuridae ω_t_=1** | 64 | -3949.76 | ω_t_=1.0000, ω_0_= 0.0906 |
| **Emballonuridae and Pteropodidae** | 65 | -3941.43 | ω_tm_=0.0888, ω_0_=0.0938 |
| **Emballonuridae and Pteropodidae ω_tm_=1** | 64 | -3972.20 | ω_tm_=1.0000, ω_0_=0.0904 |
| **Branch-site models** | | | |
| **Pteropodidae** | 67 | -3873.76 | site class 0 1 2a 2b  proportion 0.90997 0.09003 0.00000 0.00000  background ω 0.04729 1.00000 0.04729 1.00000  foreground ω 0.04729 1.00000 1.00000 1.00000 |
| **Pteropodidae ω_m_=1** | 66 | -3873.76 | site class 0 1 2a 2b  proportion 0.90997 0.09003 0.00000 0.00000  background ω 0.04729 1.00000 0.04729 1.00000  foreground ω 0.04729 1.00000 1.00000 1.00000 |
| **Emballonuridae** | 67 | -3873.76 | site class 0 1 2a 2b  proportion 0.90997 0.09003 0.00000 0.00000  background ω 0.04729 1.00000 0.04729 1.00000  foreground ω 0.04729 1.00000 1.00000 1.00000 |
| **Emballonuridae ω_t_=1** | 66 | -3873.76 | site class 0 1 2a 2b  proportion 0.90997 0.09003 0.00000 0.00000  background ω 0.04729 1.00000 0.04729 1.00000  foreground ω 0.04729 1.00000 1.00000 1.00000 |
| **Emballonuridae and Pteropodidae** | 67 | -3873.76 | site class 0 1 2a 2b  proportion 0.90997 0.09003 0.00000 0.00000  background ω 0.04729 1.00000 0.04729 1.00000  foreground ω 0.04729 1.00000 1.00000 1.00000 |
| **Emballonuridae and Pteropodidae ω_tm_=1** | 66 | -3873.76 | site class 0 1 2a 2b  proportion 0.90997 0.09003 0.00000 0.00000  background ω 0.04729 1.00000 0.04729 1.00000  foreground ω 0.04729 1.00000 1.00000 1.00000 |
