# Supplementary material for: Multiple Episodes of Convergence in Genes of the Dim Light Vision Pathway in Bats
Source: PLoS One. 2012 Apr 11;7(4):e34564. doi: 10.1371/journal.pone.0034564 (PMC3324491; doi:10.1371/journal.pone.0034564)
Supplement: Table S3 — Primers used for amplifying and sequencing CRX genes in bats. (DOC) [file pone.0034564.s009.doc]

Table S3: Primers used for amplifying and sequencing *CRX* genes in bats.

| **Primer name** | **Primer sequence (5' to 3')** | **Ta (℃)** |
| --- | --- | --- |
| **RNA primers** | | |
| CRX_S24 | gcc CCA CTA YTC CgT CAR Cg | Touchdown 60℃-50℃ |
| CRX_A881 | CAg gCA CTC TgA TCY TTg Tag TCC |  |
| **DNA primers** | | |
| CRX_S1 | CCC CAC TAT TCC gTC ARC gC | 63℃ |
| CRX_A2500 | CAg gCA CTC TgA TCY TTg TAg TC |  |
| **Sequencing primers** | | |
| MX -1-A108 | gTg gCC CTg ATg TCA YCC |  |
| MX -2-A274 | CAg gCA TgT TAg Agg ARg AA |  |
| MX-3-S341 | gCC TCT gCT TTC TgC TCT TCC C |  |
| MX-3-A362 | ggg AAg AgC AgA AAg CAg Agg C |  |
| CRX_S810 | CgR gAg Cgg ACC ACC TTY AC |  |
| CRX_A914 | YTT BAg VgC CAC CTC CTC |  |
| CRX_A2220 | AAR CRg Mgg CTg ggg CgT Ag |  |
| CRX_A1897 | CAY TTA gCC CTR Cgg TTC TT |  |
| CRX_S1877 | CAA gAA CCg YAg RgC YAA RTg |  |
| CRX_A2443 | gTg CCT gTR ggg TCY TTR AA |  |
| CRX_S1875 | TTC AAg AAC CgY AgR gCY AA |  |
| CRX_A2500 | CAg gCA CTC TgA TCY TTg TAg TC |  |
