# Supplementary material for: Multiple Episodes of Convergence in Genes of the Dim Light Vision Pathway in Bats
Source: PLoS One. 2012 Apr 11;7(4):e34564. doi: 10.1371/journal.pone.0034564 (PMC3324491; doi:10.1371/journal.pone.0034564)
Supplement: Table S4 — Primers used for amplifying and sequencing SAG genes in bats. (DOC) [file pone.0034564.s010.doc]

Table S4: Primers used for amplifying and sequencing *SAG* genes in bats.

| **Primer name** | **Primer sequence (5' to 3')** | **Ta (℃)** |
| --- | --- | --- |
| **RNA primers** | | |
| S16070 | CAA gTA gAg CCT gTg gAY gg | Touchdown 60℃-50℃ |
| A16071 | CAC CTT gAT CTg gTA AgA CAC C |  |
| **DNA primers** | | |
| S18392 | GAG GCT ATT TTC AAG GAC | Touchdown 60℃-50℃ |
| A18393 | CTA Cgg CCA ggA AgA CAT |  |
| **Sequencing primers** | | |
| 16070 | CAA gTA gAg CCT gTg gAY gg |  |
| 16071 | CAC CTT gAT CTg gTA AgA CAC C |  |
| 18392 | GAG GCT ATT TTC AAG GAC |  |
| 18393 | CTA Cgg CCA ggA AgA CAT |  |
