# Supplementary material for: Multiple Episodes of Convergence in Genes of the Dim Light Vision Pathway in Bats
Source: PLoS One. 2012 Apr 11;7(4):e34564. doi: 10.1371/journal.pone.0034564 (PMC3324491; doi:10.1371/journal.pone.0034564)
Supplement: Table S5 — Species and their accession numbers of CRX and SAG genes used in this research. (DOC) [file pone.0034564.s011.doc]

Table S5: Species and their accession numbers of *CRX* and *SAG* genes used in this research.

| order | family | Species | *CRX* gene | *SAG* gene |
| --- | --- | --- | --- | --- |
| Primates | Hominidae | *Homo sapiens* | ENSG00000105392 | ENSG00000130561 |
| Primates | Hominidae | *Pan troglodytes* | ENSPTRG00000011217 | ENSPTRG00000013052 |
| Primates | Cercopithecidae | *Macaca mulatta* | ENSMMUG00000021902 | ENSMMUG00000023359 |
| Ruminantia | Bovidae | *Bos taurus* | ENSBTAG00000021185 | ENSBTAG00000021480 |
| Carnivora | Canidae | *Canis familiaris* | ENSCAFG00000004068 | ENSCAFG00000011811 |
| Rodentia | Muridae | *Mus musculus* | ENSMUSG00000041578 | ENSMUSG00000056055 |
| Rodentia | Muridae | *Rattus norvegicus* | ENSRNOG00000013890 | ENSRNOG00000018185 |
| Chiroptera | Emballonuridae | *Cormura brevirostris* | HQ651134 |  |
| Chiroptera | Emballonuridae | *Saccopteryx bilineata* | HQ651140 |  |
| Chiroptera | Emballonuridae | *Taphozous melanopogon* | HQ651124,HQ651125 | JF831437 |
| Chiroptera | Molossidae | *Chaerephon plicatus* | HQ651131 | JF831434 |
| Chiroptera | Mormoopidae | *Pteronotus parnellii* | HQ651138 |  |
| Chiroptera | Noctilionidae | *Noctilio leporinus* | HQ651132 |  |
| Chiroptera | Phyllostomidae | *Artibeus lituratus* | HQ651139 |  |
| Chiroptera | Phyllostomidae | *Glossophaga soricina* | HQ651141 |  |
| Chiroptera | Phyllostomidae | *Lonchophylla thomasi* | HQ651137 |  |
| Chiroptera | Phyllostomidae | *Micronycteris nicefori* | HQ651136 |  |
| Chiroptera | Phyllostomidae | *Sturnira lilium* | HQ651135 |  |
| Chiroptera | Pteropodidae | *Cynopterus sphinx* | HQ651143,HQ651144 | JF831433 |
| Chiroptera | Pteropodidae | *Eonycteris spelaea* | HQ651107 | JF831422 |
| Chiroptera | Pteropodidae | *Rousettus leschenaultii* | HQ651145,HQ651146 |  |
| Chiroptera | Pteropodidae | *Rousettus leschenaultii* | HQ651149 | JF831430 |
| Chiroptera | Pteropodidae | *Sphaerias blanfordi* | HQ651142 |  |
| Chiroptera | Rhinolophidae | *Hipposideros armiger* | HQ651094 | JF831428 |
| Chiroptera | Rhinolophidae | *Hipposideros armiger* | HQ651122,HQ651123 | JF831438 |
| Chiroptera | Rhinolophidae | *Hipposideros larvatus* | HQ651118,HQ651119 | JF831440 |
| Chiroptera | Rhinolophidae | *Hipposideros pomona* | HQ651120,HQ651121 | JF831439 |
| Chiroptera | Rhinolophidae | *Hipposideros pomona* | HQ651148 | JF831431 |
| Chiroptera | Rhinolophidae | *Rhinolophus marshalli* | HQ651147 | JF831432 |
| Chiroptera | Rhinolophidae | *Rhinolophus pearsonii* | HQ651110,HQ651111 | JF831445 |
| Chiroptera | Rhinolophidae | *Rhinolophus pusillus* | HQ651112,HQ651113 | JF831443 |
| Chiroptera | Rhinolophidae | *Rhinolophus pusillus* | HQ651114,HQ651115 | JF831442 |
| Chiroptera | Rhinolophidae | *Rhinolophus sinicus* | HQ651104,HQ651105,HQ651106 | JF831423 |
| Chiroptera | Rhinolophidae | *Rhinolophus sinicus* | HQ651095,HQ651096 | JF831427 |
| Chiroptera | Rhinolophidae | *Rhinolophus sinicus* | HQ651097,HQ651098 |  |
| Chiroptera | Rhinolophidae | *Rhinolophus sinicus* | HQ651102,HQ651103 | JF831424 |
| Chiroptera | Rhinolophidae | *Rhinophylla pumilio* | HQ651133 |  |
| Chiroptera | Vespertilionidae | *Miniopterus fuliginosus* |  | JF831429 |
| Chiroptera | Vespertilionidae | *Miniopterus fuliginosus* |  | JF831444 |
| Chiroptera | Vespertilionidae | *Myotis davidii* | HQ651099,HQ651100 | JF831426 |
| Chiroptera | Vespertilionidae | *Myotis laniger* | HQ651101 | JF831425 |
| Chiroptera | Vespertilionidae | *Myotis laniger* | HQ651116,HQ651117 | JF831441 |
| Chiroptera | Vespertilionidae | *Myotis pilosus* | HQ651126 | JF831436 |
| Chiroptera | Vespertilionidae | *Nyctalus plancyi* | HQ651127,HQ651128 | JF831435 |
| Chiroptera | Vespertilionidae | *Nyctalus plancyi* | HQ651129,HQ651130 |  |
| Chiroptera | Vespertilionidae | *Tylonycteris pachypus* | HQ651108 | JF831446 |
| Chiroptera | Vespertilionidae | *Tylonycteris robustula* | HQ651109 |  |
